# The Interaction between Fuzzy Subsets and Groupoids

**DOI:** 10.1155/2014/246285

**Published:** 2014-04-09

**Authors:** Seung Joon Shin, Hee Sik Kim, J. Neggers

**Affiliations:** ^1^Department of Physics, University of Michigan, Ann Arbor, MI 48109, USA; ^2^Department of Mathematics, Research Institute for Natural Sciences, Hanyang University, Seoul 133-791, Republic of Korea; ^3^Department of Mathematics, University of Alabama, Tuscaloosa, AL 35487-0350, USA

## Abstract

We discuss properties of a class of real-valued functions on a set *X* ^2^ constructed as finite (real) linear combinations of functions denoted as [(*X*, *∗*); **μ**], where (*X*, *∗*) is a groupoid (binary system) and **μ** is a fuzzy subset of *X* and where [(*X*, *∗*); **μ**](*x*, *y*): = **μ**(*x∗y*) − min*⁡*{**μ**(*x*), **μ**(*y*)}. Many properties, for example, **μ** being a fuzzy subgroupoid of *X*, *∗*), can be restated as some properties of [(*X*, *∗*); **μ**]. Thus, the context provided opens up ways to consider well-known concepts in a new light, with new ways to prove known results as well as to provide new questions and new results. Among these are identifications of many subsemigroups and left ideals of (Bin(*X*), □) for example.

## 1. Introduction

The notion of a fuzzy subset of a set was introduced by Zadeh [1]. His seminal paper in 1965 has opened up new insights and applications in a wide range of scientific fields. Rosenfeld [2] used the notion of a fuzzy subset to set down corner stone papers in several areas of mathematics. Mordeson and Malik [3] published a remarkable book,* Fuzzy Commutative Algebra*, presented a fuzzy ideal theory of commutative rings, and applied the results to the solution of fuzzy intersection equations. The book included all the important work that has been done on *L*-subspaces of a vector space and on *L*-subfields of a field.

Kim and Neggers [4] introduced the notion of Bin(*X*) and obtained a semigroup structure. Fayoumi [5] introduced the notion of the center *Z*Bin(*X*) in the semigroup Bin(*X*) of all binary systems on a set *X* and showed that a groupoid (*X*, ·) ∈ *Z*Bin(*X*) if and only if it is a locally zero groupoid. The present authors [6] introduced the notion of abelian fuzzy subgroupoids on a groupoid and discuss diagonal symmetric relations, convex sets, and fuzzy centers on Bin(*X*).

In this paper, we discuss properties of a class of real valued functions on a set *X* ^2^ constructed as finite (real) linear combinations of functions denoted [(*X*, ∗); *μ*], where (*X*, ∗) is a groupoid (binary system) and *μ* is a fuzzy subset of *X* and where [(*X*, ∗); *μ*](*x*, *y*): = *μ*(*x*∗*y*) − min⁡{*μ*(*x*), *μ*(*y*)}. Many properties, for example, *μ* being a fuzzy subgroupoid of (*X*, ∗), can be restated as some properties of [(*X*, ∗); *μ*]. Thus, the context provided opens up ways to consider well-known concepts in a new light, with new ways to prove known results as well as to provide new questions and new results. Among these are identifications of many subsemigroups and left ideals of (Bin(*X*), □), for example.

## 2. Preliminaries

Given a nonempty set *X*, we let Bin(*X*) denote the collection of all groupoids (*X*, ∗), where ∗ : *X* × *X* → *X* is a map and where ∗(*x*, *y*) is written in the usual product form. Given elements (*X*, ∗) and (*X*, ·) of Bin(*X*), define a product “□” on these groupoids as follows:

(1) $$\begin{array}{c} \left(X,*\right)□\left(X,\cdot \right)=\left(X,□\right), \end{array}$$

where

(2) $$\begin{array}{c} x□y=\left(x*y\right)\cdot \left(y*x\right) \end{array}$$

for any *x*, *y* ∈ *X*. Using that notion, Kim and Neggers proved the following theorem.

**Theorem 1 (see [4])**

( Bin (*X*), □) is a semigroup; that is, operation “□” as defined in general is associative. Furthermore, the left zero semigroup is the identity for this operation.

## 3. Interactions

Given a set *X*, one may consider (i) a fuzzy subset *μ* of *X*, that is, a mapping *μ* : *X* → [0,1], and (ii) a groupoid (binary system) (*X*, ∗), where ∗ : *X* ^2^ → *X* is a mapping. It is thus possible to consider a fuzzy subgroupoid of a groupoid as a composition [(*X*, ∗); *μ*](*x*, *y*):(*X*, ∗)→[0,1], where *μ* and (*X*, ∗) interact to satisfy the condition:

(3) $$\begin{array}{c} \mu \left(x*y\right)-\min\left\{\mu \left(x\right),\mu \left(y\right)\right\}\geq 0 \end{array}$$

for all *x*, *y* ∈ *X*.

One might easily conceive related notions such as “an almost fuzzy subgroupoid of a fuzzy groupoid,” which though here unspecified will be considered in what follows. Since “almost” itself is a typical “fuzzy math” notion, we will consider* the intersection between a fuzzy subset μ and a groupoid *(*X*, ∗) as given by the function denoted by [(*X*, ∗); *μ*] : *X* ^2^ → **R**, where

(4) [(X,∗);μ](x,y):=μ(x∗y)−min⁡{μ(x),μ(y)}

for all *x*, *y* ∈ *X*.

Note that intersection [(*X*, ∗); *μ*] need not be a fuzzy subset of (*X*, ∗).

**Example 2**

Let *X* be set **R** of all real numbers and let + be the usual addition on **R**. Define a map *μ* : *X* → [0,1] by *μ*(*x*): = *x* − ⌊*x*⌋. Then [(*X*, +); *μ*](*x*, *y*) = *μ*(*x* + *y*) − min⁡{*μ*(*x*), *μ*(*y*)} = (*x* + *y*) − ⌊*x* + *y*⌋ − min⁡{*x* − ⌊*x*⌋, *y* − ⌊*y*⌋}. If we let *x* = *y* = 1/2, then [(*X*, +); *μ*](1/2, 1/2) = −1/2. Hence, [(*X*, +); *μ*] is not a fuzzy subset of (*X*, +).

**Example 3**

Let *X* be set **R** of all real numbers. Define a binary operation ∗ on **R** by *x*∗*y* : = *x* − ⌊*x*⌋. Then *μ*(*x*∗*y*) = *μ*(*x* − ⌊*x*⌋) = (*x* − ⌊*x*⌋) − ⌊*x* − ⌊*x*⌋⌋ = *x* − ⌊*x*⌋ = *μ*(*x*) for all *x*, *y* ∈ *X*. It follows that [(*X*, ∗); *μ*](*x*, *y*) = *μ*(*x*∗*y*) − min⁡{*μ*(*x*), *μ*(*y*)} = *μ*(*x*) − min⁡{*μ*(*x*), *μ*(*y*)} ≥ 0. Hence, [(*X*, ∗); *μ*] is a fuzzy subset of (*X*, ∗).

**Example 4**

Let *X* be set **R** of all real numbers, and let + be the usual addition on **R** and let *x*∗*y* : = *x* − ⌊*x*⌋ for all *x*, *y* ∈ **R**. Define (*X*, □): = (*X*, ∗)□(*X*, +). Then *x*□*y* = (*x*∗*y*)+(*y*∗*x*) = *x* − ⌊*x*⌋ + *y* − ⌊*y*⌋. It follows that *μ*(*x*□*y*) = *x* + *y* − (⌊*x*⌋ + ⌊*y*⌋) − ⌊*x* + *y* − (⌊*x*⌋ + ⌊*y*⌋)⌋ for all *x*, *y* ∈ *X*. Assume that *x* − ⌊*x*⌋ = *y* − ⌊*y*⌋ = 0.6. Then *μ*(*x*□*y*) = 1.2 − 1 = 0.2 and hence [(*X*, □); *μ*](*x*, *y*) = *μ*(*x*□*y*) − min⁡{*μ*(*x*), *μ*(*y*)} = −0.4. Hence, [(*X*, □); *μ*] is not a fuzzy subgroupoid of (*X*, □).

Given an intersection [(*X*, ∗); *μ*], note that the smallest value obtainable is when *μ*(*x*∗*y*) = 0 and *μ*(*x*) = *μ*(*y*) = 1. If such a pair (*x*, *y*) exists, then [(*X*, ∗); *μ*](*x*, *y*) = *μ*(*x*∗*y*) − min⁡⁡{*μ*(*x*), *μ*(*y*)} = −1. We call such a pair (*x*, *y*) a *μ*-*orthogonal pair* on (*X*, ∗).

**Example 5**

Let **R** be the set of all real numbers, and let (**R**, ∗) be the groupoid defined by $x*y:=x+y\sqrt{2}$, for all *x*, *y* ∈ **R**. Let *μ* : **R** → [0,1] be the characteristic function of the rationals; that is, if *x* is rational, then *μ*(*x*) = *χ*_**Q**_(*x*) = 1 and if *x* is irrational, then *μ*(*x*) = 0. Suppose that *x* and *y* ≠ 0 are both rational. Then $x*y=x+y\sqrt{2}$ is irrational. In that case, we have [(*X*, ∗); *μ*](*x*, *y*) = *μ*(*x*∗*y*) − min⁡{*μ*(*x*), *μ*(*y*)} = 0 − 1 = −1. Thus, (*x*, *y*) is an *χ*_**Q**_-orthogonal pair on (**R**, ∗).

**Proposition 6**

Given (*X*, ∗) ∈ Bin (*X*), if *μ* is a fuzzy subalgebra of (*X*, ∗), then there is no pair (*x*, *y*) ∈ *X* ^2^ such that (*x*, *y*) is a *μ*-orthogonal pair on (*X*, ∗).

**Proof**

If *μ* is a fuzzy subalgebra of (*X*, ∗), then *μ*(*x*∗*y*) ≥ min⁡{*μ*(*x*), *μ*(*y*)} for all *x*, *y* ∈ *X*. Assume that (*x*, *y*) is a *μ*-orthogonal pair on (*X*, ∗). Then *μ*(*x*∗*y*) − min⁡{*μ*(*x*), *μ*(*y*)} = −1. It follows that 0 ≤ *μ*(*x*∗*y*) = min⁡{*μ*(*x*), *μ*(*y*)} − 1 ≤ 0, which shows that min⁡{*μ*(*x*), *μ*(*y*)} = 1. Since *μ* is a fuzzy subalgebra of (*X*, ∗), we obtain *μ*(*x*∗*y*) = *μ*(*x*) = *μ*(*y*) = 1. Hence, 0 = *μ*(*x*∗*y*) − min⁡{*μ*(*x*), *μ*(*y*)} = −1, a contradiction.

Given an intersection [(*X*, ∗); *μ*], a pair (*x*, *y*) ∈ *X* ^2^ is said to be a *μ*-*parallel pair* on (*X*, ∗) if [(*X*, ∗); *μ*](*x*, *y*) = 1. In Example 5, $\left(3,4\sqrt{2}\right)$ is a *μ*-parallel pair on (**R**, ∗). In fact, $\left[\left(X,*\right);\mu \right]\left(3,4\sqrt{2}\right)=\mu \left(3*4\sqrt{2}\right)-\min\left\{\mu \left(3\right),\mu \left(4\sqrt{2}\right)\right\}=\mu \left(11\right)-\min\left\{\mu \left(3\right),\mu (4\sqrt{2})\right\}=1-\min\{1,0\}=1$.

Given an element *α* ∈ **R** and an interaction [(*X*, ∗); *μ*], we define *α*[(*X*, ∗); *μ*] by

(5) (α[(X,∗);μ])(x,y):=α[μ(x∗y)−min⁡{μ(x),μ(y)}]

for all *x*, *y* ∈ *X*. Using these notions, we will consider that a generating set for a vector space of groupoids (*X*, ∗_*i*_) ∈ Bin(*X*) can be expressed as finite sums ∑_*i*=1_^*n*^ *λ*_*i*_[(*X*, ∗_*i*_); *μ*_*i*_], where *λ*_*i*_ ∈ **R** for *i* = 1,…, *n*, and *μ*_*i*_ : (*X*, ∗_*i*_)→[0,1] is a fuzzy subset of (*X*, ∗_*i*_) and where (*α*[(*X*, ∗_*i*_); *μ*_*i*_])(*x*, *y*): = *α*[*μ*_*i*_(*x*∗_*i*_ *y*) − min⁡{*μ*_*i*_(*x*), *μ*_*i*_(*y*)}] as usual for real-valued functions. If we let *S* : = ∑_*i*=1_^*n*^ *λ*_*i*_[(*X*, ∗_*i*_); *μ*_*i*_], then −∑_*i*=1_^*n*^ | *λ*_*i*_ | ≤*S*(*x*, *y*) ≤ ∑_*i*=1_^*n*^ | *λ*_*i*_| for all *x*, *y* ∈ *X*. Given a fuzzy subset *μ* of (*X*, ∗), we define a map 1 − *μ* : *X* → [0,1] by (1 − *μ*)(*x*): = 1 − *μ*(*x*) for all *x* ∈ *X*.

**Proposition 7**

Given (*X*, ∗) ∈ Bin (*X*), if *μ* is a fuzzy subset of (*X*, ∗), then

(6) ([(X,∗);μ]+[(X,∗);1−μ])(x,y)=|μ(x)−μ(y)|

for all *x*, *y* ∈ *X*.

**Proof**

Let *S*[∗, *μ*]: = [(*X*, ∗); *μ*]+[(*X*, ∗); 1 − *μ*]. Then, for all *x*, *y* ∈ *X*, we have

(7) S[∗,μ](x,y) =μ(x∗y)−min⁡{μ(x),μ(y)}+(1−μ)(x∗y)  −min⁡{(1−μ)(x),(1−μ)(y)} =μ(x∗y)−min⁡⁡{μ(x),μ(y)}+1−μ(x∗y)  −min⁡{1−μ(x),1−μ(y)} =−min⁡{μ(x),μ(y)}+1−1+max⁡{μ(x),μ(y)} =|μ(x)−μ(y)|.

Note that such an *S*[∗, *μ*] in Proposition 7 is independent of any groupoid (*X*, ∗) ∈ Bin(*X*).

**Corollary 8**

Given (*X*, ∗), (*X*, ·) ∈ Bin (*X*), we have

(8) $$\begin{array}{c} \left[\left(X,*\right);\mu \right]+\left[\left(X,*\right);1-\mu \right]=\left[\left(X,\cdot \right);\mu \right]+\left[\left(X,\cdot \right);1-\mu \right]. \end{array}$$

**Proof**

It follows immediately from Proposition 7.

**Theorem 9**

Given (*X*, ∗) ∈ Bin (*X*) and a fuzzy subset *μ* : *X* → [0,1], there exist a groupoid (*X*, ⋆) ∈ Bin (*X*) and a fuzzy subset *ν* : *X* → [0,1] such that [(*X*, ⋆); *ν*](*x*, *y*) = max⁡{*μ*(*x*), *μ*(*y*)} − min⁡{*ν*(*x*), *ν*(*y*)} for all *x*, *y* ∈ *X*.

**Proof**

Given (*X*, ∗) ∈ Bin(*X*) and a fuzzy subset *μ* : *X* → [0,1], we define a surjective map *ν* : *X* → *Im*⁡(*μ*). Since max⁡{*μ*(*x*), *μ*(*y*)} ∈ *Im*⁡(*μ*), *ν* ^−1^(max⁡{*μ*(*x*), *μ*(*y*)}) ≠ *∅*. Define a binary operation ⋆ on *X* as follows: for any *x*, *y* ∈ *X*, *x*⋆*y* : = *z* for some *z* ∈ *ν* ^−1^(max⁡{*μ*(*x*), *μ*(*y*)}. It follows that *ν*(*x*⋆*y*) = max⁡{*μ*(*x*), *μ*(*y*)}. This means that [(*X*, ⋆); *ν*](*x*, *y*) = *ν*(*x*⋆*y*) − min⁡{*ν*(*x*), *ν*(*y*)} = max⁡{*μ*(*x*), *μ*(*y*)} − min⁡{*ν*(*x*), *ν*(*y*)}.

**Corollary 10**

Given (*X*, ∗) ∈ Bin (*X*) and a fuzzy subset *μ* : *X* → [0,1], there exists a groupoid (*X*, ⋆) ∈ Bin (*X*) such that [(*X*, ⋆); *μ*] = *S*[∗, *μ*].

**Proof**

In the proof of Theorem 9, if we let *ν* : = *μ*, then [(*X*, ⋆); *ν*](*x*, *y*) = max⁡{*μ*(*x*), *μ*(*y*)} − min⁡{*μ*(*x*), *μ*(*y*)} = |*μ*(*x*) − *μ*(*y*)| = *S*[∗, *μ*](*x*, *y*) by Proposition 7.

## 4. Composition of Interactions

Given a groupoid (*X*, ∗) ∈ Bin(*X*), we define a set *F*(*X*): = {*μ* | *μ* : *X* → [0,1]:a  map}. We consider the composition *μ*□*ν* of fuzzy subsets *μ*, *ν* ∈ *F*(*X*) as a function *φ*(*x*) whose variables are *μ*(*x*) and *ν*(*x*); that is, (*μ*□*ν*)(*x*): = *φ*(*μ*(*x*), *ν*(*x*)). For example, (*μ*□*ν*)(*x*) = (1/2)(*μ*(*x*) + *ν*(*x*)),(*μν*)(*x*) = *λμ*(*x*) + (1 − *λ*)*ν*(*x*), *λ* ∈ [0,1], or $(\mu □\nu )(x)=\sqrt{\mu (x)\nu (x)}$.

In general, (*μ*□*ν*)□*ρ* ≠ *μ*□(*ν*□*ρ*). In fact, if we define $(\mu □\nu )(x):=\sqrt{\mu (x)\nu (x)}$, then $[(\mu □\nu )□\rho ](x)=\sqrt{\sqrt{\mu (x)\nu (x)}\rho (x)}$, while [*μ*□(*ν*□*ρ*)](*x*)$=\sqrt{\mu (x)\sqrt{\nu (x)\rho (x)}}$. A fuzzy subset *μ* ∈ *F*(*X*) is said to be □-*idempotent* if *μ*□*μ* = *μ*.

Given interactions [(*X*, ∗); *μ*], [(*X*, ·); *ν*], we define a new interaction as follows:

(9) $$\begin{array}{c} \left[\left(X,□\right);\mu □\nu \right]:=\left[\left(X,*\right);\mu \right]□\left[\left(X,\cdot \right);\nu \right], \end{array}$$

where (*X*, □) = (*X*, ∗)□(*X*, ·) and (*μ*□*ν*)(*x*): = *φ*(*μ*(*x*), *ν*(*x*)) for all *x* ∈ *X*.

It is easy to see that if (*μ*□*ν*)□*ρ* = *μ*□(*ν*□*ρ*), then ([(*X*, ∗); *μ*]□[(*X*, ·); *ν*])□[(*X*, ⋆); *ρ*] = [(*X*, ∗); *μ*]□([(*X*, ·); *ν*]□[(*X*, ⋆); *ρ*] for any (*X*, ∗),(*X*, ·), (*X*, ⋆) ∈ Bin(*X*).

**Example 11**

Given a groupoid (*X*, ∗) ∈ Bin(*X*) and *μ*, *ν* ∈ *F*(*X*), we define *μ*□*ν* : = (1/2)(*μ* + *ν*). It follows that

(10) ([(X,∗);μ]□[(X,∗);ν])(x,y) =[(X,∗)□(X,∗);μ□ν](x,y) =(μ□ν)(x□y)−min⁡{(μ□ν)(x),(μ□ν)(y)} =12{μ(x□y)+ν(x□y)}  −min⁡{12(μ(x)+ν(x)),12(μ(y)+ν(y))} =12{μ((x∗y)∗(y∗x))+ν((x∗y)∗(y∗x))}  −min⁡{12(μ(x)+ν(x)),12(μ(y)+ν(y))}.

Given an interaction [(*X*, ∗); *μ*], we have already seen that −1 ≤ [(*X*, ∗); *μ*] ≤ 1. If 0 ≤ [(*X*, ∗); *μ*] ≤ 1, then *μ* is a fuzzy subgroupoid of (*X*, ∗) and conversely. Thus, we will be interested in bounds *α* ≤ [(*X*, ∗); *μ*] ≤ *β* as classification parameters of the [(*X*, ∗); *μ*]. Hence, we usually take *α* : = inf⁡{[(*X*, ∗); *μ*](*x*, *y*) | *x*, *y* ∈ *X*} and *β* : = sup⁡{[(*X*, ∗); *μ*](*x*, *y*) | *x*, *y* ∈ *X*} for the “narrowest” fit.

**Theorem 12**

Given *μ* ∈ *F*(*X*) and (*X*, ∗), (*X*, ·) ∈ Bin (*X*), if the interactions [(*X*, ∗); *μ*], [(*X*, ·); *μ*] have the following bounds: *α* ≤ [(*X*, ∗); *μ*] ≤ *β*, *γ* ≤ [(*X*, ·); *μ*] ≤ *δ*, then the interaction [(*X*, ∗)□(*X*, ·); *μ*] has the bound *α* + *γ* ≤ [(*X*, ∗)□(*X*, ·); *μ*] ≤ *β* + *δ*.

**Proof**

Given *x*, *y* ∈ *X*, we let *p* : = min⁡{*μ*(*x*∗*y*), *μ*(*y*∗*x*)} and *q* : = min⁡{*μ*(*x*), *μ*(*y*)}. If *α* ≤ [(*X*, ∗); *μ*] ≤ *β*, *γ* ≤ [(*X*, ·); *μ*] ≤ *δ*, then *μ*((*x*∗*y*) · (*y*∗*x*)) − min⁡{*μ*(*x*∗*y*), *μ*(*y*∗*x*)}∈[*γ*, *δ*]. Since *μ*(*x*∗*y*) − min⁡{*μ*(*x*), *μ*(*y*)}∈[*α*, *β*] and *μ*(*y*∗*x*) − min⁡{*μ*(*x*), *μ*(*y*)}∈[*α*, *β*], we have *μ*(*x*∗*y*), *μ*(*y*∗*x*)∈[*α* + *q*, *β* + *q*]. It follows that *p* = min⁡{*μ*(*x*∗*y*), *μ*(*y*∗*x*)}∈[*α* + *q*, *β* + *q*]; that is, *p* − *q* ∈ [*α*, *β*]. Thus, *μ*(*x*□*y*) − min⁡{*μ*(*x*), *μ*(*y*)} = *μ*((*x*∗*y*) · (*y*∗*x*)) − *q* = *μ*((*x*∗*y*) · (*y*∗*x*)) − *p* + *p* − *q* ∈ [*γ*, *δ*]+[*α*, *β*] = [*α* + *γ*, *β* + *δ*].

Note that map *μ* in Theorem 12 need not be a fuzzy subset of ((*X*, ∗)□(*X*, ·); *μ*) if *β* + *δ* > 1.

**Theorem 13**

Let *α* ≤ [(*X*, ∗); *μ*] ≤ *β* and *γ* ≤ [(*X*, ·); *μ*] ≤ *δ*. If there exist *σ*, *τ* ∈ **R** such that |*μ*(*x*∗*y*) − *μ*(*y*∗*x*)|∈[*σ*, *τ*] for all *x*, *y* ∈ *X*, then

(11) $$\begin{array}{c} \alpha +\gamma -\tau \leq \left[\left(X,*\right)X,\cdot ;\mu \right]\leq \beta +\delta -\sigma . \end{array}$$

**Proof**

If *γ* ≤ [(*X*, ·); *μ*] ≤ *δ*, then

(12) μ(x□y)−min⁡{μ(x∗y),μ(y∗x)} =μ((x∗y)·(y∗x))  −min⁡{μ(x∗y),μ(y∗x)}∈[γ,δ]

for all *x*, *y* ∈ *X*. If *α* ≤ [(*X*, ∗); *μ*] ≤ *β*, then

(13) $$\begin{array}{c} \mu \left(x*y\right)-\min\left\{\mu \left(x\right),\mu \left(y\right)\right\}\in \left[\alpha ,\beta \right], \end{array}$$

(14) $$\begin{array}{c} \mu \left(y*x\right)-\min\left\{\mu \left(x\right),\mu \left(y\right)\right\}\in \left[\alpha ,\beta \right]. \end{array}$$

Assume that there exist *σ*, *τ* ∈ **R** such that |*μ*(*x*∗*y*) − *μ*(*y*∗*x*)|∈[*σ*, *τ*] for all *x*, *y* ∈ *X*. Let *μ*(*x*∗*y*) + *ρ* = *μ*(*y*∗*x*) for some *ρ* ≥ 0. Then *μ*(*x*∗*y*) − min⁡{*μ*(*x*), *μ*(*y*)} + *ρ* = *μ*(*y*∗*x*) − min⁡{*μ*(*x*), *μ*(*y*)} ≤ *β*. It follows that *μ*(*x*∗*y*) − min⁡{*μ*(*x*), *μ*(*y*)} ≤ *β* − *ρ* ≤ *β* − *σ*. By applying (14), we obtain

(15) $$\begin{array}{c} \min\left\{\mu \left(x*y\right),\mu \left(y*x\right)\right\}-\min\left\{\mu \left(x\right),\mu \left(y\right)\right\}\leq \beta -\sigma . \end{array}$$

Similarly, since ≤*μ*(*y*∗*x*) − min⁡{*μ*(*x*), *μ*(*y*)} = *μ*(*x*∗*y*) − min⁡{*μ*(*x*), *μ*(*y*)} + *ρ*, we obtain

(16) α−τ≤α−ρ≤μ(x∗y)−min⁡{μ(x),μ(y)}.

By applying (14), we obtain

(17) α−τ≤α−ρ≤min⁡{μ(x∗y),μ(y∗x)}−min⁡{μ(x),μ(y)}.

Using (12), (17), and (15), we obtain the following:

(18) α−τ+γ ≤μ(x□y)−min⁡{μ(x∗y),μ(y∗x)}  +min⁡{μ(x∗y),μ(y∗x)}−min⁡{μ(x),μ(y)} ≤β−σ+δ,

which shows that

(19) α−τ+γ≤[(X,□);μ](x,y)=[(X,∗)(X,·);μ]≤β−σ+δ.

Let *μ* ∈ *F*(*X*). A groupoid (*X*, ∗) ∈ Bin(*X*) is said to be *μ*-*commutative* if *μ*(*x*∗*y*) = *μ*(*y*∗*x*) for all *x*, *y* ∈ *X*.

**Proposition 14**

If (*X*, ·) ∈ Bin (*X*) is *μ*-commutative, then ((*X*, ∗)□(*X*, ·); *μ*) is also *μ*-commutative for all (*X*, ∗) ∈ Bin (*X*).

**Proof**

For all *x*, *y* ∈ *X*, we have *μ*(*x*□*y*) = *μ*((*x*∗*y*) · (*y*∗*x*)) = *μ*((*y*∗*x*) · (*x*∗*y*)) = *μ*(*y*□*x*).

Note that Proposition 14 shows that the set of all *μ*-commutative groupoids forms a left ideal of the semigroup (Bin(*X*), □).

**Theorem 15**

Given (*X*, ∗) ∈ Bin (*X*), if *μ*_*i*_ : *X* → [0,1] are fuzzy subsets of (*X*, ∗) and *λ* ∈ [0,1], then

(20) λ[(X,∗);μ1]+(1−λ)[(X,∗);μ2]  ≤[(X,∗);λμ1+(1−λ)μ2].

**Proof**

Let *μ* : = *λμ*_1_ + (1 − *λ*)*μ*_2_. Given *a*, *b*, *c*, *d* ∈ [0,1], we have min⁡{*a* + *b*, *c* + *d*} ≥ min⁡{*a*, *c*} + min⁡{*c*, *d*}. It follows that, for all *x*, *y* ∈ *X*,

(21) [(X,∗);μ](x,y) =μ(x∗y)−min⁡{μ(x),μ(y)} =[λμ1+(1−λ)μ2](x∗y)  −min⁡{[λμ1+(1−λ)μ2](x),[λμ1+(1−λ)μ2](y)} =λμ1(x∗y)+(1−λ)μ2(x∗y)  −min⁡{λμ1(x)+(1−λ)μ2(x), λμ1(y)+(1−λ)μ2(y)} ≥λ[μ1(x∗y)−min⁡{μ1(x),μ1(y)}]  +(1−λ)[μ2(x∗y)−min⁡{μ2(x),μ2(y)}] =λ[(X,∗);μ1](x∗y)+(1−λ)[(X,∗);μ2](x∗y),

proving the theorem.

## 5. Representable Functions by Interactions

A function *f* : *X* ^2^ → [−1,1] is said to be* representable* if *f* can be represented as [(*X*, ∗); *μ*] for some groupoid (*X*, ∗) ∈ Bin(*X*) and a fuzzy subset *μ* : *X* → [0,1]. We denote it by *f* = [(*X*, ∗); *μ*].

Let {*a*_*n*_}_*n*=1_^*∞*^ be a sequence of real numbers such that, for any *n* and *k*,

(22) $$\begin{array}{c} -\frac{A}{k}\leq \frac{1}{k}\left[a_{n+1}+\cdots +a_{n+k}\right]\leq \frac{B}{k} \end{array}$$

for some real numbers *A*, *B* > 0. Then {*a*_*n*_}_*n*=1_^*∞*^ is called a* special sequence* of type (*A*, *B*).

We have the following observations.

1. If *A*′ ≥ *A* and *B*′ ≥ *B*, then a special sequence of type (*A*, *B*) is also a special sequence of type (*A*′, *B*′).
2. If {*a*_*n*_}_*n*=1_^*∞*^ is a special sequence of type (*A*, *B*) and {*b*_*n*_}_*n*=1_^*∞*^ is a special sequence of type (*C*, *D*), then, for real numbers *λ*, *δ* > 0, {*λa*_*n*_ + *δb*_*n*_}_*n*=1_^*∞*^ is a special sequence of type (*λA* + *δC*, *λB* + *δD*). If *A* = *B* = *C* = *D* = 1 and *λ* + *δ* = 1, then *λA* + *δC* = 1 and *λB* + *δD* = 1. Such a special sequence of type (1,1) is called a* standard special sequence.*
3. If *b*_*n*_ = *a*_*n*+*t*_, *t* ≥ 0 and if {*a*_*n*_}_*n*=1_^*∞*^ is a special sequence of type (*A*, *B*), then {*b*_*n*_}_*n*=1_^*∞*^ is also a special sequence of type (*A*, *B*). It follows that if {*a*_*n*_}_*n*=1_^*∞*^ is a special sequence, then, for all *n* ≥ 1, lim⁡_*k*→*∞*_(*a*_*n*+1_ + ⋯+*a*_*n*+*k*_)/*k* = 0.
4. Notice that if {*a*_*n*_ = *α*}_*n*=1_^*∞*^, then lim⁡_*k*→*∞*_(*a*_*n*+1_ + ⋯+*a*_*n*+*k*_)/*k* = lim⁡_*k*→*∞*_(*kα*/*k*) = *α*, whence, *α* ≠ 0 implies that {*a*_*n*_ = *α*}_*n*=1_^*∞*^ is not a special sequence.
5. Notice that lim⁡_*k*→*∞*_[1/(*n* + 1) + ⋯+1/(*n* + *k*)]/*k* = 0, since lim⁡_*k*→*∞*_(1/*k*)∫_*n*_^*n*+*k*^(*dx*/*x*) = lim⁡_*k*→*∞*_[ln⁡(1 + *k*/*n*)/1/*k*] = 0. However, there is no *B* such that, for any *n* and *k*, [1/(*n* + 1) + ⋯+1/(*n* + *k*)]/*k* ≤ *B*/*k*; that is, 1/(*n* + 1) + ⋯+1/(*n* + *k*) ≤ *B* since ∑_*n*=1_^*∞*^(1/*n*) diverges. Hence, the sequence {*a*_*n*_ = 1/*n*}_*n*=1_^*∞*^ is not special for any pair (*A*, *B*), with *A*, *B* > 0, a pair of real numbers.

Let (*X*, ∗) be a groupoid. For *x*_1_ ∈ *X*, determine a sequence *d*(*x*_1_): = {*x*_1_, *x*_2_,…, *x*_*n*_,…} as follows: *x*_2_ = *x*_1_∗*x*_1_,  *x*_3_ = *x*_2_∗*x*_2_,…, *x*_*n*+1_ = *x*_*n*_∗*x*_*n*_,….. We consider *d*(*x*_1_) to be a* doubling sequence for x*_1_ *relative to *(*X*, ∗).

**Theorem**

If *f* = [(*X*, ∗); *μ*], then the doubling sequence must be a standard special sequence.

**Proof**

Let *f* = [(*X*, ∗); *μ*] for some groupoid (*X*, ∗) and a fuzzy subset *μ* : *X* → [0,1]. If *d*(*x*_1_) is a doubling sequence in (*X*, ∗), then

(23) ∑i=1nf(xi,xi) =∑i=1n[μ(xi∗xi)−μ(xi)] =[μ(xn+1)−μ(xn)]+[μ(xn)−μ(xn−1)]  +⋯+[μ(x3)−μ(x2)]+[μ(x2)−μ(x1)] =μ(xn+1)−μ(x1).

Since −1 ≤ *μ*(*x*_*n*+1_) − *μ*(*x*_1_) ≤ 1, we obtain −1/*n* ≤ (1/*n*)[∑_*i*=1_^*n*^ *f*(*x*_*i*_, *x*_*i*_)] ≤ 1/*n*. Replace *x*_1_ by *x*_*k*+1_,…, *x*_*n*_ by *x*_*n*+*k*_, the same inequalities are obtained. Hence, we conclude that {*d*(*x*_1_)} is a standard special sequence.

**Corollary 17**

If *f*(*x*, *y*) ≥ *α* > 0 or *f*(*x*, *y*) ≤ *β* < 0, then *f*(*x*, *y*) is not representable as *f* = [(*X*, ∗); *μ*].

**Proof**

If *f*(*x*, *y*) ≥ *α* > 0, then, for any expression [*f*(*x*_*n*+1_, *x*_*n*+1_)+⋯+*f*(*x*_*n*+*k*_, *x*_*n*+*k*_]/*k*, we obtain a value in excess of *kα*/*k* = *α*, and thus it is not possible to obtain a special sequence of type (*A*, *B*) for any finite *B*, since then lim⁡_*k*→*∞*_(*B*/*k*) ≥ *α* > 0 also.

**Example 18**

Let *X* : = [0, *∞*) and let *x*∗*y* : = (*x* + *y*)/(2 + *x* + *y*) for *x*, *y* ∈ *X*. Then (*X*, ∗) is a groupoid. Assume that a mapping *f* : *X* ^2^ → **R** is defined by *f*(*x*, *y*): = (*x* + *y*)/(2 + *x* + *y*), ∀*x*, *y* ∈ *X*, then *f*(*x*, *x*) = *x*/(*x* + 1). If we let *x*_1_ : = 1, then *f*(1,1) = 1/2, *f*(1/2, 1/2) = 1/3,…, *f*(1/*n*, 1/*n*); then the doubling sequence *d*(*f*(1,1)) = {1/*n*} is not a standard special sequence. By Theorem 16, the mapping *f* cannot be representable by the groupoid (*X*, ∗) and any fuzzy subset *μ* of *X*.

**Problem**

Let *X* : = **R** be the set of all real numbers. Define a function **S** **i** **n** : *X* ^2^ → **R** by **S** **i** **n**(*x*, *y*): = sin(*x* + *y*), the sine function, for all *x*, *y* ∈ **R**. The problem is to decide whether or not the mapping **S** **i** **n** is representable.

If we assume that **S** **i** **n** = [(*X*, ∗); *μ*] for some groupoid (*X*, ∗) ∈ Bin(*X*) and a fuzzy subset *μ* : *X* → [0,1], then we may deduce many properties of the representation. Note that *μ*(*π*/4) = 0 and *μ*((*π*/4)∗(*π*/4)) = 1. In fact, **S** **i** **n**(*π*/4, *π*/4) = sin(*π*/2) = *μ*((*π*/4)∗(*π*/4)) − *μ*(*π*/4) = 1. Much other information is available via the use of trigonometric properties. Thus, for example, **S** **i** **n**(*x*, *y* + *π*) = sin(*x* + *y* + *π*) = −sin(*x* + *y*) = −**S** **i** **n**(*x*, *y*) = −[*μ*(*x*∗*y*) − min⁡{*μ*(*x*), *μ*(*y*)}] = *μ*(*x*∗(*y* + *π*)) − min⁡{*μ*(*x*), *μ*(*y* + *π*)] so that *μ*(*x*∗(*y* + *π*)) + *μ*(*x*∗*y*) = min⁡{*μ*(*x*), *μ*(*y* + *π*)} + min⁡{*μ*(*x*), *μ*(*y*)}. Thus, for example, *μ*(*x*) = 1 implies *μ*(*x*∗(*y* + *π*)) + *μ*(*x*∗*y*) = *μ*(*y* + *π*) + *μ*(*y*) and *μ*(*x*) = 0 implies *μ*(*x*∗(*y* + *π*)) = −*μ*(*x*∗*y*), whence *μ*(*x*∗*y*) = 0 for any *y* whatsoever. Hence, if Ker⁡*μ* = *μ* ^−1^(0), then Ker⁡*μ* is a right ideal of (*X*, ∗).

In the sine function case, doubling sequences may generate sequences with positive and negative values whose average over sections [*f*(*x*_*n*+1_, *x*_*n*+1_)+⋯+*f*(*x*_*n*+*k*_, *x*_*n*+*k*_)]/*k* must behave properly to yield at least the possibility of a representation.

Here is another example of a negative solution to the representation problem. Suppose that {(*x*_*n*_, *y*_*n*_)}_*n*=1_^*∞*^ is a sequence of elements in *X* ^2^ and that *f* : *X* ^2^ → [−1,1] has a sign pattern on {*f*(*x*_*n*_, *y*_*n*_)}_*n*=1_^*∞*^ of the following type {+, −, +, +, −, −, +, +, +, −, −, −, +, +, +, +,…} and that *f*(*x*_*n*_, *x*_*n*_) ≥ *α* > 0 when it is positive. If *f* is representable, that is, *f* = [(*X*, ∗); *μ*], and if {*f*(*x*_*n*_, *y*_*n*_)}_*n*=1_^*∞*^ is a doubling sequence, then for *n* = *k*(*k* + 1), there are *k* slots of positive value between *i* = *n* and *i* = *n* + *k*. Thus, summing over all these slots, one obtains an upper bound of value *k* (the number of slots). Another associated value is the average of the functional values of that segment from *i* = *n* to *i* = *n* + *k*, and thus at least *α* > 0. This violates the condition that the doubling sequence obtained from *f* = [(*X*, ∗); *μ*] must be a standard special sequence. Hence, *f* is not representable.

**Problem**

Given *f*(*x*, *y*), if it is believed/known that *f* = [(*X*, ∗); *μ*], develop a method of finding a groupoid (*X*, ∗) and a fuzzy subset *μ* such that *f* = [(*X*, ∗); *μ*]. So far, the best results have been of the negative kind, but interesting nevertheless.

As a closing comment, we observe that the class of representable functions *f* : *X* ^2^ → [−1,1] is quite enormous with common properties to be examined. The doubling sequence technique developed above is an example of such a common property which can be applied to the existence problem of representations for function of the type addressed here.
